# Enucleation in a Cownose Ray* (Rhinoptera bonasus)*

**DOI:** 10.1155/2018/5048948

**Published:** 2018-03-19

**Authors:** A. Abraham Gabriel, S. T. Yee-Nin, Lawan Adamu, H. M. D. Hassan, A. H. Wahid

**Affiliations:** ^1^Department of Farm & Exotic Animals Medicine & Surgery, Faculty of Veterinary Medicine, Universiti Putra Malaysia (UPM), 43400 Serdang, Selangor, Malaysia; ^2^Department of Veterinary Clinical Studies, Faculty of Veterinary Medicine, Universiti Putra Malaysia (UPM), 43400 Serdang, Selangor, Malaysia; ^3^Department of Veterinary Medicine, Faculty of Veterinary Medicine, University of Maiduguri, PMB 1069, Borno State, Nigeria

## Abstract

Trauma is a common problem in Cownose Ray during mating season in both wild and captive rays. Enucleation is indicated when there is an ocular trauma. A 5-year-old female Cownose Ray* (Rhinoptera bonasus)* from Aquaria of Kuala Lumpur Convention Centre (KLCC) was presented to University Veterinary Hospital (UVH), Universiti Putra Malaysia, with a complaint of protruding left eye, which resulted from crushing into artificial coral during mating season. There were a hyphema in the traumatic left eye, periorbital tissue tear, exposed left eye socket, and multiple abrasions on both pectoral fins. The Cownose was anaesthetized and maintained with isoeugenol and on-field emergency enucleation of the left eye was performed. It was managed medically with postoperative enrofloxacin, tobramycin ointment, and povidone iodine. No suture breakdown and secondary infection were observed at day 7 after enucleation during revisit. At day 24 after enucleation, the Cownose responded well to treatment with excellent healing progression and no surgical complication was observed.

## 1. Introduction

The Cownose Ray* (Rhinoptera bonasus)* is also known as Cowfish or Skeete. They are classified as “Near Threaten” due to low productivity and as bycatch with pounds net. Schools of Cownose Ray usually occurred in estuaries, in shore, and in open ocean and can be found in Western Atlantic Ocean, Gulp of Mexico, and part of Caribbean Sea [1]. They gain their name from their unique forehead which resembled the nose of cattle [2]. Cownose Ray is one of the 42 species in the Myliobatidae family under the Elasmobranchii order and the cartilaginous fish including sharks and skates. As carnivores, their diet is consisted with bottom-dwelling shellfish, lobsters, carbs, and fish. Electroreceptors on snout are an excellent sense to touch and smell aids them to locate their prey [3].

Mating behaviors can be observed in the wild from early October to late June but primarily between April and June, when male-inflicted bites wounds on female pectoral fins are commonly observed [4]. Trauma is also a common problem in captive rays during breeding season, including ocular trauma, and nonfatal external injuries. Furthermore, there is a likelihood of internal injuries which sometimes causes death [5]. Therefore, the objective of the case report is to treat the ocular trauma by adopting a surgical approach such as enucleation to accomplish the desired response.

## 2. Case Report

A 5-year-old female Cownose* (Rhinoptera bonasus)* was managed in Oceanarium, the large scale exhibition area of Aquaria in Kuala Lumpur (KLCC) with over 40 species of elasmobranchs and teleost fish. She is fed with marine chopped fish twice a day and Mazuri supplement (vitamins and minerals).

Cownose's caretaker noticed her left eye ball is protruding out from the orbit. He suspected she had crushed into the artificial coral when the male rays chased her for mating, and the period correlates with the mating season. The case was presented to University Veterinary Hospital (UVH), Universiti Putra Malaysia, on the same day that he complained of the eye trauma during field visit to Aquaria KLCC.

**Figure 1:**
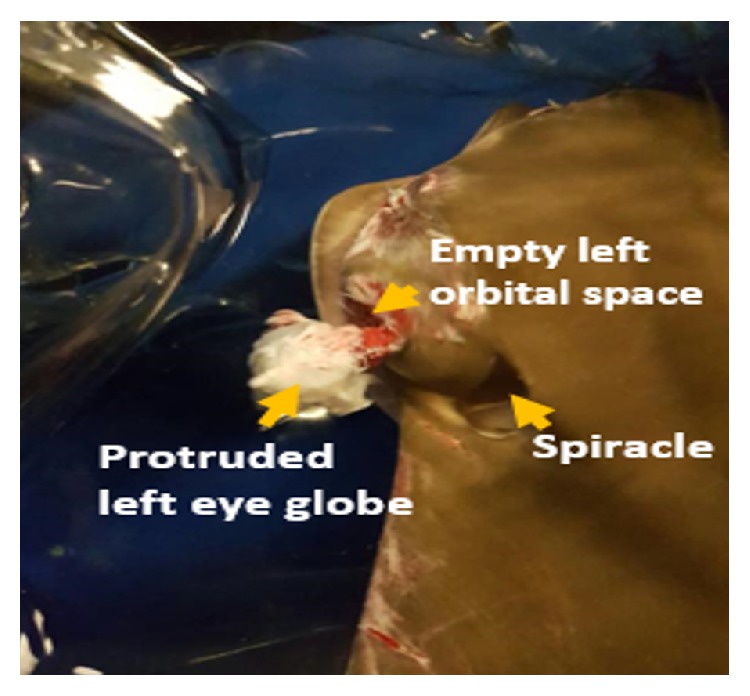
Protrusion of left eye globe from its orbit attached to the optic nerve with its adjacent connective tissue, indicating exposed left eye orbital space and teared periocular tissue. Hyphema cannot be viewed from this angle.

**Figure 2:**
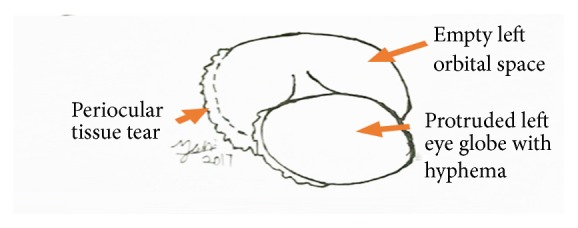
Schematic diagram of the ocular trauma.

Physical examination was carried out. The Cownose was alert and responsive as she was actively swimming in the holding area. She was weighing 8 kg and had a Body Condition Score of 3 out of 5 and less than 5% dehydration. Temperature and pulse rate were not obtained, while the respiratory rate was 65 breaths per minutes. The left eye globe with hyphema was protruded from the orbital space and attached to the optic nerve (Figure 1). The pupillary reflex could not be assessed due to hyphema. The left orbital space was exposed and periocular tissue tear was present around the left orbit (Figure 2). Besides, multiple abrasions were observed at the cranial margin of the both pectoral fins, which was more severe on left fin.

Immersion anaesthesia was opted. Seventeen ppm of isoeugenol (brand name: Aqui-s) was used as anaesthetic agent for both induction and maintenance of surgical plane. The anaesthetic bath was prepared by filling a tank with 475 L of artificial sea water from the holding area and added with 14.8 mL of Aqui-s. Ventilation rate was the only parameter that was used to monitor the anaesthesia depth of the Cownose. The normal ventilation rate was taken at rest before induction, which will be used as the baseline to monitor ventilation throughout the anaesthesia [6]. Ventilation rate of Cownose was taken by observing movement of spiracular flap and recorded every 5 minutes.

**Figure 3:**
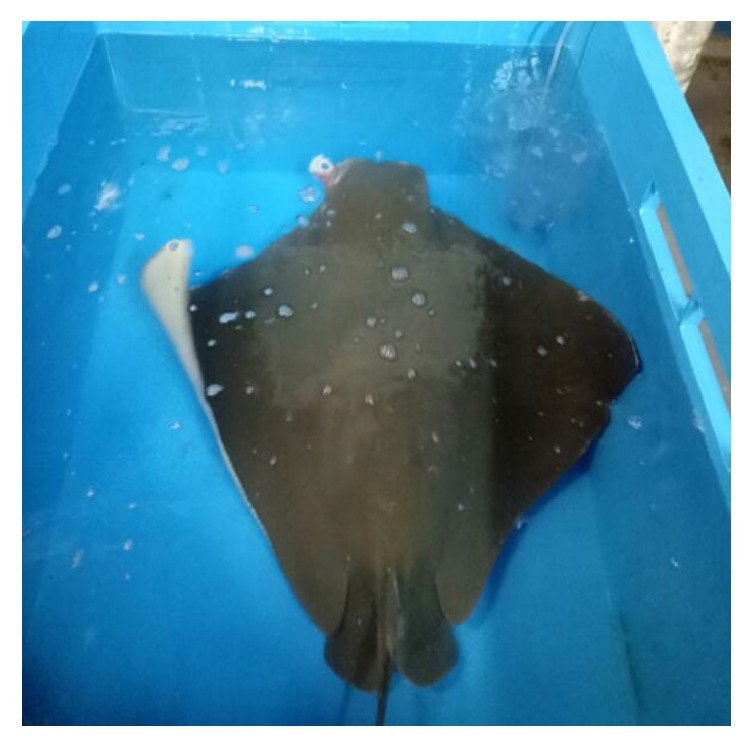
Cownose in the induction tank that contains 17 ppm of isoeugenol.

**Figure 4:**
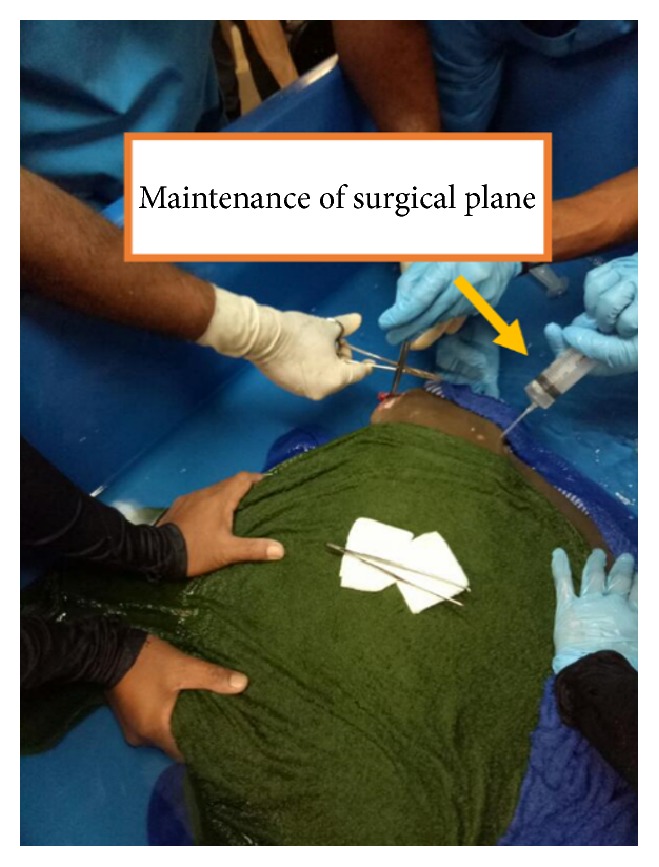
Maintenance of surgical plane was achieved by continuously flushing marine water that contains 17 ppm isoeugenol through spiracle by using a 20 mL syringe.

The Cownose was left in the anaesthetic bath for 10 minutes until its ventilation rate ceased to 40 breaths per minute and dropped in pectoral fin stroke activity (Figure 3). Later she was transferred by using canvas hammock to surgical tank with wet towel at the bottom and covered with another wet towel on her dorsum to maintain skin moisture [7] (Figure 4). The maintenance of surgical plane and establishment of rebreathing system was done by flushing water with anaesthetics agent through the gills via right spiracle (respiratory opening caudal to eye) with a 20 mL syringe.

The Cownose was presented in ventral recumbency and assistants aided in restraining by gently exerting force at the dorsum as a part of tonic immobilization. Routine skin preparation around the left eye was done by using diluted chlorhexidine gluconate (dilution ratio 1 : 30) and diluted povidone iodine (dilution ratio 1 : 30) [7].

**Figure 5:**
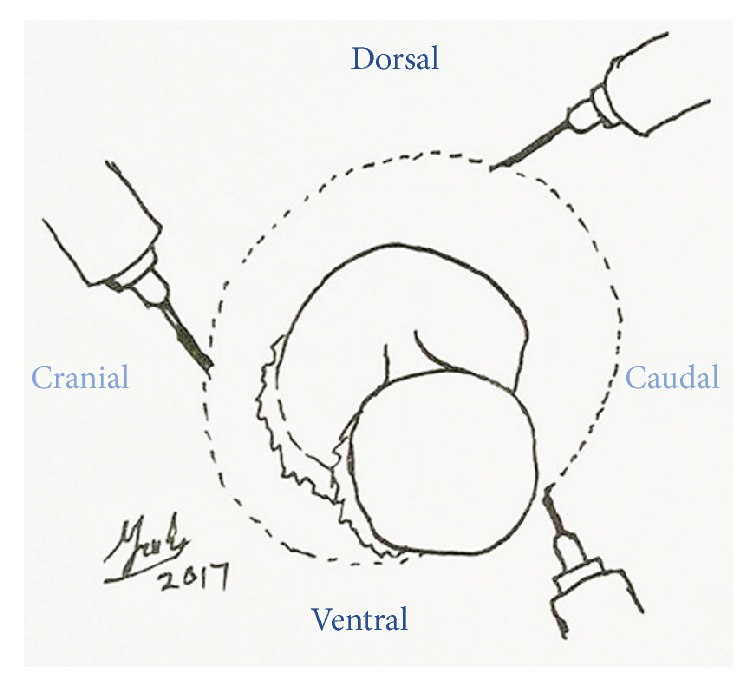
A ring block was made by using 5 mL of lidocaine to infiltrate and desensitize the periocular skin. This served as local block as well as short term local analgesia.

**Figure 6:**
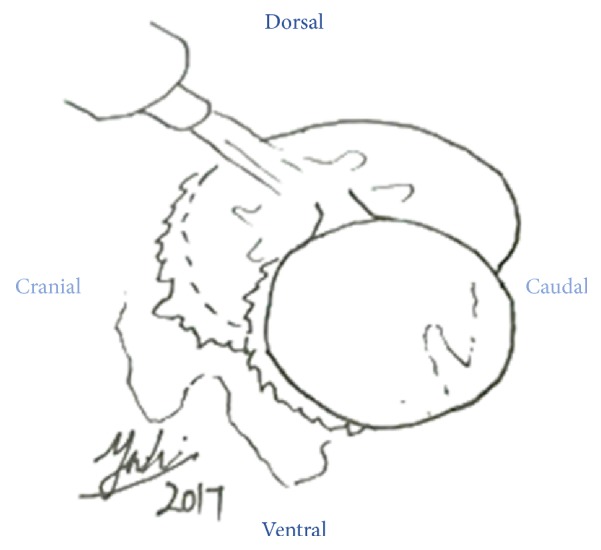
Diluted gentamycin was used to flush the orbital space and globe to reduce contamination.

**Figure 7:**
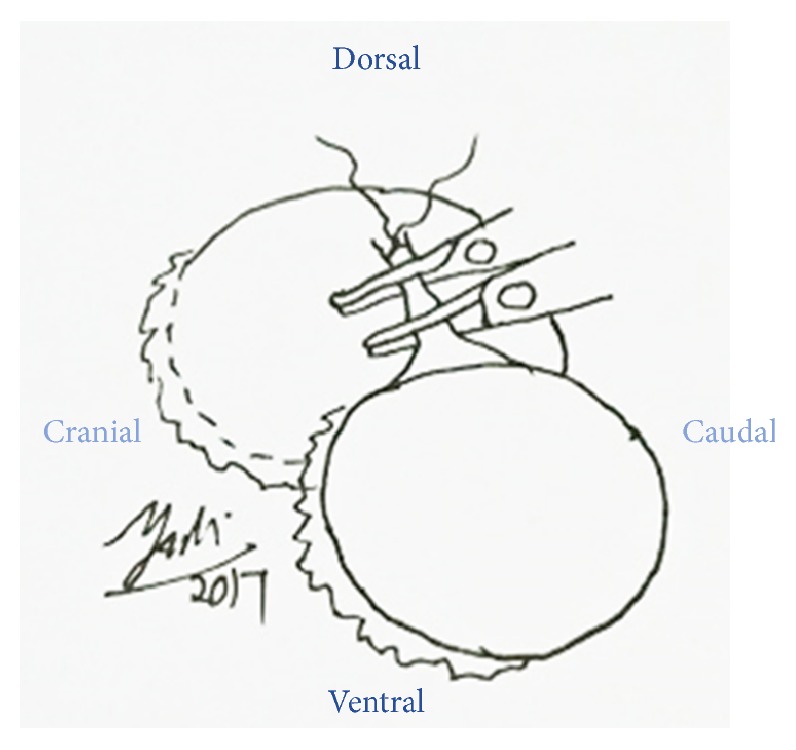
2 Rochester Pean forceps were placed on the optic nerve, while ligation was done proximal to first forceps for haemostasis.

**Figure 8:**
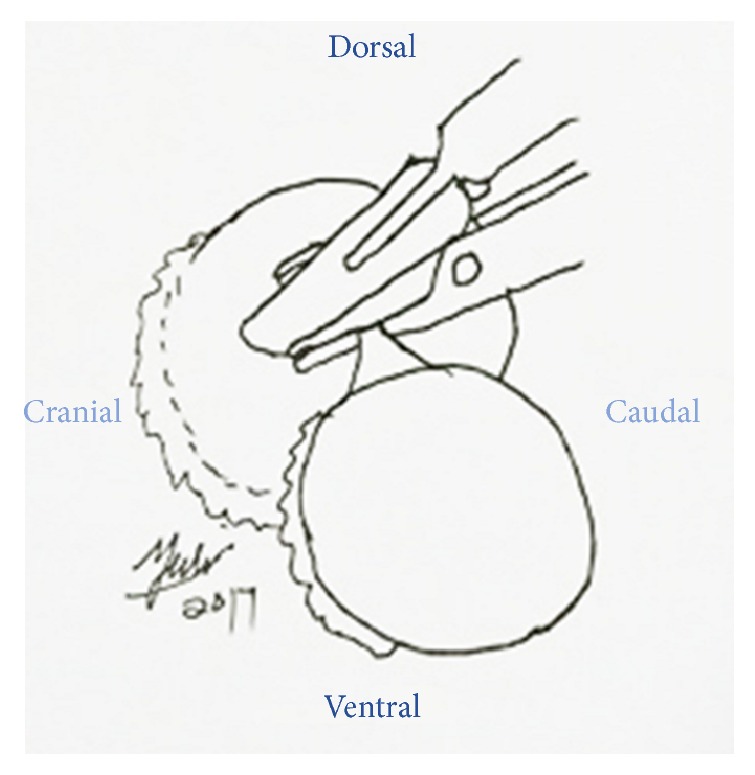
The optic nerve was transected between the Rochester Pean forceps by using scalpel blade #20.

**Figure 9:**
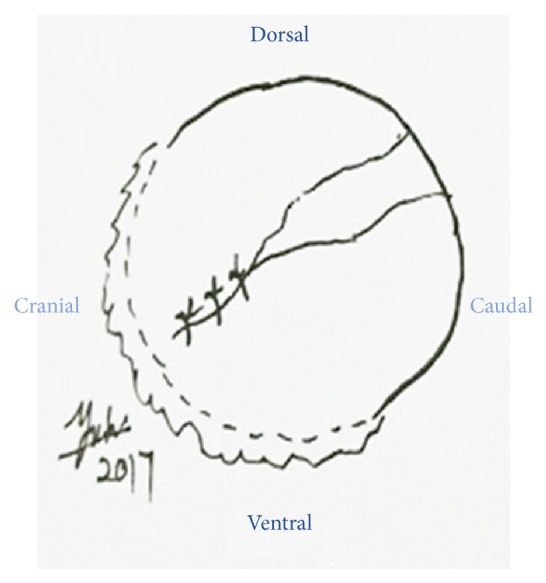
Inner and outer muscular layer were sutured by 2.0 PDS in interrupted suture pattern.

**Figure 10:**
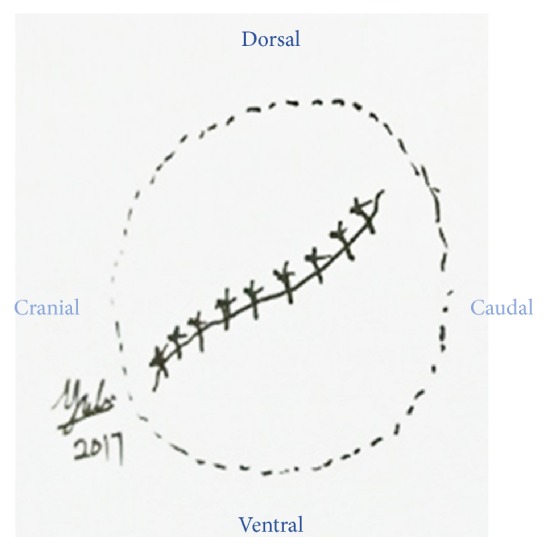
Periocular skin closure was done by 2.0 PDS in interrupted suture pattern.

A ring block was done around the left eye by using 5 mL 20 mg/mL Lidocaine as local anaesthesia to reversibly desensitize the skin as well as analgesia (Figure 5). Diluted gentamycin (5 mL, 100 mg/mL gentamycin + 5 mL sterile water) was prepared to flush the left orbital space and globe to minimize secondary infection since sterile surgical field is impossible to be established in the field setting (Figure 6). The optic nerve was clamped by using 2 Rochester Pean forceps (Figure 7). A surgeon knot was placed proximal to the first forceps with 2.0 PDS to ligate the blood vessel for haemostasis. Then the optic nerve was transected between the 2 forceps by using scalpel blade #20 (Figure 8). The globe, the remaining conjunctivae fat, and extraocular muscle tissue were removed. The pedicle was checked for presence of bleeding before releasing it into the orbital space. The inner and outer muscular attachments were sutured with 2.0 PDS using interrupted suture pattern to close the orbital space (Figure 9). Periorbital skin closure was done with 2.0 PDS in interrupted suture pattern (Figure 10).

The enucleation procedure took 15 minutes to be completed. Fresh sea water without an anaesthetic agent was used to flush through the spiracle to perfuse the gills and accelerate recovery from anaesthesia. The recovery from anaesthesia was indicated by increasing ventilation rate to preinduction rate, which was 66 bpm in Cownose and when she started struggling actively by flapping pectoral fins at 25th minutes.

Postoperatively, 5 mg/kg enrofloxacin was administered intramuscularly at the dorsolateral musculature close to the spine and terramycin ointment (active component: oxytetracycline hydrochloride) was applied twice a day topically on the suture site to prevent secondary infection of the suture site, orbital space, and optic sulcus [8]. Besides, the caretaker was instructed to apply povidone iodine (brand name: Betadine) on the abrasions of the pectoral fins twice a day until wound healed to prevent the proliferation of* Fusarium sp*., an environment saprophytic fungi [8–10].

Client education consisted of isolating the Cownose from the school in holding area until surgical wound healed before releasing it to the large exhibition area, for ease of monitoring and for the prevention of possible infection at suture site as well as for administering daily treatment [11]. Besides, daily monitoring of suture site for break and possible signs of secondary infection such as discolouration of skin, fungal growth on the wound, inappetence, and loss of body condition is recommended by Mylniczenko and Penfold [5]. Next, putting of the mating pairs into separate tanks during mating season is necessary and a good practice. The mating process of rays is always aggressive as female rays always are being bitten and injured by the male rays, in which death can occur in severe case [12]. Others include reducing risk of crushing into decorations; isolation also reduces the stress of mating animals by avoiding being attacked by other fish during and after mating.

**Figure 11:**
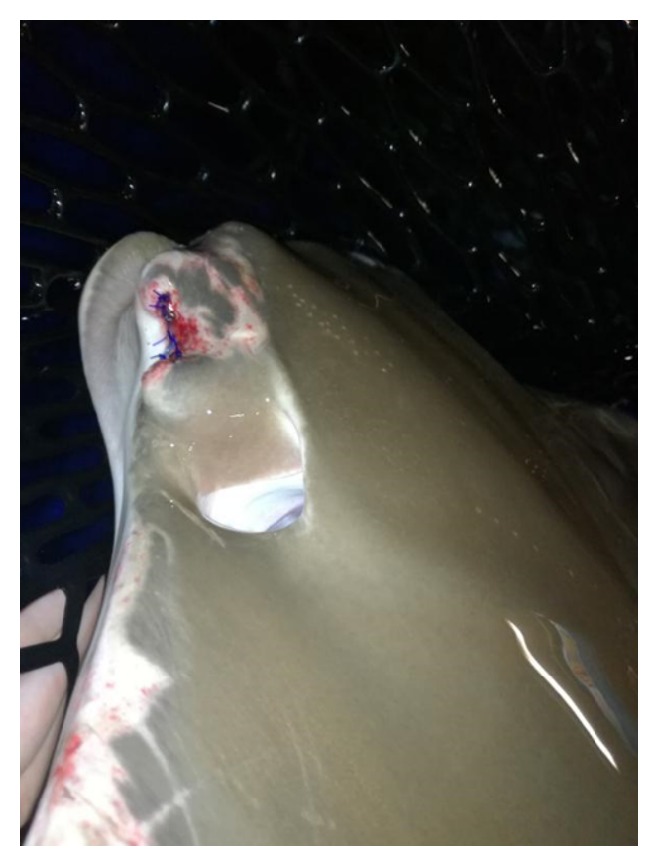
Day 7 after enucleation. There was no suture break, no skin discolouration, and no fungal growth on the surgical wound. Besides, the abrasion on the left cranial margin of pectoral fin was healing well with white fibrous scar tissue. According to the caretaker, she was having good appetite after operation.

At day 7 after operation, we had a revisit trip to Aquaria KLCC for the follow-up. She was responding well to the treatment with good appetite and no clinical abnormalities were observed, and suture materials were still intact (Figure 11). The last follow-up was on day 24 after operation and was done by phone; Cownose was having good appetite and the scars were returning to normal skin colour.

## 3. Discussion

According to Mylniczenko and Penfold [5], trauma is a common problem in ray which occurred due to aggressive mating behaviour. The male-inflicted injuries such as ocular damage, fin damage, and ulceration of ventral skin could be commonly observed during mating season. A study done on reproductive biology of Cownose Ray in wild at Charlotte Harbor Estuarine System, Florida, divulged the presence of bite wound and scars on pectoral fins of female Cownose Ray which can be observed between April and June as well as observation of group of male rays chasing female rays for mating [4]. However, no ocular damage was reported from the study. Besides, ocular trauma due to blunt trauma can be seen in Cownose rays. This is due to the lack of anatomical structures for protection of the globe, such as partially exposed globe from the cranium and absence of eyelids [13]. In the present case report, Cownose was kept in a captive environment with a lot of artificial decorations and obstacles. Therefore she could be potentially crushed into these objects when chased by male rays. In short, ocular damage due to blunt trauma is possible in an aquarium setting.

A safe and effective anaesthetic protocol can contribute to a successful surgical outcome [7]. Injectable anaesthesia such as ketamine [14] can be employed in sharks. Advantage of injectable anaesthesia was to allow the capture of animal in large exhibits, but no injectable anaesthesia was reported in rays [13]. There were also a few choices of immersion anaesthesia agent reported to be used in rays [15], while the usage of MS-222 is the most common [16, 17]. However, MS-222 is acidic in aqueous solution with pH of approximate 3 and must be evaluated in marine system before induction although salt water contains adequate inherent buffering [18]. Eugenol (Clove oil) can result in rapid induction and prolonged recovery when compared to MS-222 but has a narrow margin of safety which may be seen as respiratory failure in higher dose [7]. Isoeugenol (brand name: Aqui-S) which is structurally similar to eugenol was effective at low concentration compared to eugenol and has less adverse reaction [18], although it will caused impaired ventilation and cardiovascular depression [19]. In the present case report, we selected isoeugenol because it was relatively easier to be prepared in an on-field operation and caused less stress to Cownose. Throughout the anaesthesia, although we observed mild reduction of ventilation rate, no any detrimental effect was observed in the Cownose. Therefore, isoeugenol is safe and can be considered as a good choice for immersion anaesthesia of Cownose Ray.

The administration of postoperative antibiotics is controversial as in mammalian patient and shall not be a replacement for poor aseptic techniques due to emerging of antimicrobial resistance reported in many species [18]. However, the justification for the present case was the limitation to establish an aseptic surgical field in such an on-field situation. In addition, our patient was for exhibition purpose but not for human consumption. By referring to Elasmobranch Husbandry Manual that was published by Ohio Biological Survey 2004, a few drug of choice had been described. Bath is a common technique but medicated water must be disposed carefully in accordance with regulations; meanwhile some oral medications can be rejected due to the unusual taste [20]. Parental administration (intramuscular injection) was suitable in the present case report because it can be performed during the recovery period, and this procedure ensures that the animal received the correct dosage and reduces drug wastage. Enrofloxacin, the quinolone class, was selected because it has a broad spectrum activity against most gram-negative and many gram-positive bacteria. Besides, it was reported not to have an adverse effect on cartilage and its half-life in the plasma is 114 hours after injection probably due to its slow drug metabolism and excretion in the elasmobranch [15].

Vision does play an important role in most of the predatory behaviour, which is associated with the presence of a binocular vision [11]. However in the Cownose's case, the removal of the left eye would affect her predatory behaviour. Rays had a poor vision as compared to other senses such as olfactory and taste [5]. From the anatomical aspect, ray's eyes were positioned on the dorsal side, which is the opposite of their mouth that is located at the ventral side. Therefore, they must rely on the senses other than vision in foraging [21]. Besides, rays did possess an anterior binocular convergence but the overlapping area was much less compared to other predators. This implicated that ray's vision was not the primary sensory organ that aided in hunting [17]. Lastly, there is one powerful sensory adaptation present in all elasmobranch that consisted of multiple small subcutaneous vesicles located around the head, snout, or mouth area. The ampullae of Lorenzini allowed elasmobranchs to detect electric fields from a prey and response to mechanical stimuli, even if vision and olfactory cues were absent [22]. Ocular damage can occur in a female Cownose Ray during mating season in aquarium setting resulting from avoidance from being mated by aggressive male rays, limited space, presence of plenty artificial decorations, and anatomical structure of the forehead [5, 13]. Enucleation is the best option for case of severe ocular trauma if the traumatized eye was not viable [7]. This procedure also yielded a good outcome because impaired vision was not necessary to affect predatory behaviour of a Cownose Ray [17]. Next, judicious antibiotics administration in ray is crucial as a part of postoperative management to prevent secondary wound infection [7]. Finally, management of mating animals during mating season is the key of reducing mating behaviour associated mortality in captive environment [23].

## 4. Conclusion

In conclusion, enucleation did not compromise the hunting behaviour and the patient's quality of life. Therefore, surgical approach such as enucleation has the desired efficacy in the present case report.
